# Exploring the relationship between intron retention and chromatin accessibility in plants

**DOI:** 10.1186/s12864-017-4393-z

**Published:** 2018-01-05

**Authors:** Fahad Ullah, Michael Hamilton, Anireddy S.N. Reddy, Asa Ben-Hur

**Affiliations:** 1 Computer Science Department, Colorado State University, 1873 Campus Delivery, Fort Collins, 80523 CO USA; 2 Department of Biology, Colorado State University, 1878 Campus Delivery, Fort Collins, 80523 CO USA

**Keywords:** Intron retention, Chromatin accessibility, DNase I hypersensitive sites

## Abstract

**Background:**

Intron retention (IR) is the most prevalent form of alternative splicing in plants. IR, like other forms of alternative splicing, has an important role in increasing gene product diversity and regulating transcript functionality. Splicing is known to occur co-transcriptionally and is influenced by the speed of transcription which in turn, is affected by chromatin structure. It follows that chromatin structure may have an important role in the regulation of splicing, and there is preliminary evidence in metazoans to suggest that this is indeed the case; however, nothing is known about the role of chromatin structure in regulating IR in plants. DNase I-seq is a useful experimental tool for genome-wide interrogation of chromatin accessibility, providing information on regions of chromatin with very high likelihood of cleavage by the enzyme DNase I, known as DNase I Hypersensitive Sites (DHSs). While it is well-established that promoter regions are highly accessible and are over-represented with DHSs, not much is known about DHSs in the bodies of genes, and their relationship to splicing in general, and IR in particular.

**Results:**

In this study we use publicly available DNase I-seq data in arabidopsis and rice to investigate the relationship between IR and chromatin structure. We find that IR events are highly enriched in DHSs in both species. This implies that chromatin is more open in retained introns, which is consistent with a kinetic model of the process whereby higher speeds of transcription in those regions give less time for the spliceosomal machinery to recognize and splice out those introns co-transcriptionally. The more open chromatin in IR can also be the result of regulation mediated by DNA-binding proteins. To test this, we performed an exhaustive search for footprints left by DNA-binding proteins that are associated with IR. We identified several hundred short sequence elements that exhibit footprints in their DNase I-seq coverage, the telltale sign for binding events of a regulatory protein, protecting its binding site from cleavage by DNase I. A highly significant fraction of those sequence elements are conserved between arabidopsis and rice, a strong indication of their functional importance.

**Conclusions:**

In this study we have established an association between IR and chromatin accessibility, and presented a mechanistic hypothesis that explains the observed association from the perspective of the co-transcriptional nature of splicing. Furthermore, we identified conserved sequence elements for DNA-binding proteins that affect splicing.

**Electronic supplementary material:**

The online version of this article (10.1186/s12864-017-4393-z) contains supplementary material, which is available to authorized users.

## Background

Alternative Splicing (AS) is a regulatory phenomenon that allows a gene to generate multiple transcripts, and has important roles in an organism’s development, growth, and response to stress [1, 2]. Recent studies using RNA-seq data show that AS is widespread in both plants and animals. The primary forms of AS are exon skipping, intron retention (IR) and alternative 5’ and 3’ site splicing. These forms of AS have different frequency of occurrence in plants and animals: exon skipping is the most prevalent form of AS in animals whereas IR is the most prevalent in plants [3]. This difference can be attributed to a number of differences in the architecture of plant and animal genes. For instance, plant introns are much shorter than those in animals. The splicing signals which are found at the 5’ and 3’ boundaries of introns, the polypyrimidine tract and the branch point sequence alone are insufficient for efficient splicing [4]. Another layer of splicing regulation occurs through Splicing Regulatory Elements (SREs), either exonic or intronic. These are binding sites for *trans*-acting splicing regulatory proteins that can either suppress or enhance splicing; SREs are known to have an important role in alternative and constitutive splicing [1, 4–6], and are usually 6- 10 nucleotides long [7].

There is an ongoing effort to understand how alternative splicing is regulated and the factors that contribute to it. Some of these factors include AU-rich and U-rich sequences in plant introns [8–10], the role of GC content in exons for efficient splicing [11], and AG-rich exonic element promoting downstream 5’ splice site selection [12]. Braunschweig et al. [13] have recently compiled a draft “splicing code”: a predictive model of IR in mammals based on around a hundred and fifty features likely to be associated with the process. SREs are an important aspect of any splicing code, and while in mammals many SREs have been experimentally identified [5, 14, 15], not much is known in plants, except for a few computationally predicted exonic splicing enhancers in arabidopsis [3, 16].

The fact that splicing can happen co-transcriptionally suggests that chromatin state is relevant for splicing [17, 18]. One of the primary tools for genome-wide exploration of chromatin is through exposure of DNA to Deoxyribonuclease I (DNAse I), which is an enzyme that cleaves DNA; sites that are sensitive to its action—DNase I hypersensitive sites (DHSs)—have been used as an indicator of regions in the DNA that are accessible *in-vivo*. DHSs have been used to identify several types of regulatory elements such as, promoters, silencers, enhancers, and insulators [19, 20]. It has been shown that when a protein binds a region of DNA, it protects it against the action of DNase I [21] and leaves a footprint which can be identified using DNase I-seq data [22, 23]. The ENCODE consortium has shown that DHSs identified in the human genome are robust markers for several genetic regulatory phenomena, including histone modifications, early replication regions, transcription factor binding sites, and transcription start sites [24].

When it comes to AS, Mercer et al. [25] have shown an association between DHSs and exon-skipping, reporting that higher numbers of DHS-containing exons are alternatively spliced. Furthermore, this study claims that DHS exons with promoter and enhancer-like features have a higher fractional overlap with AS. Specifically related to this work, the cross-talk between chromatin organization and IR has been studied in mammals [13]. They explore the co-transcriptional regulation of splicing reporting higher chromatin accessibility in retained introns and how polymerase II elongation speed affects IR and vice-versa. DNase I-seq has been used in plants [26, 27], but the data has not been analyzed in the context of AS.

Our goal is to shed light on the regulation of IR from the perspective of chromatin organization. First we test the association between DHSs and IR using DNase I seq data in arabidopsis and rice, and find that DHSs have a highly significant association with IR; we then look for evidence at the DNA level for the footprints of protein binding and find a large collection of hexamers that are conserved across arabidopsis and rice, and likely function as SREs. Finally, we discuss how these observations are consistent with current models that describe the interaction between transcription, splicing, and chromatin organization.

## Results

### DHSs are enriched in IR events

**Fig. 1:**
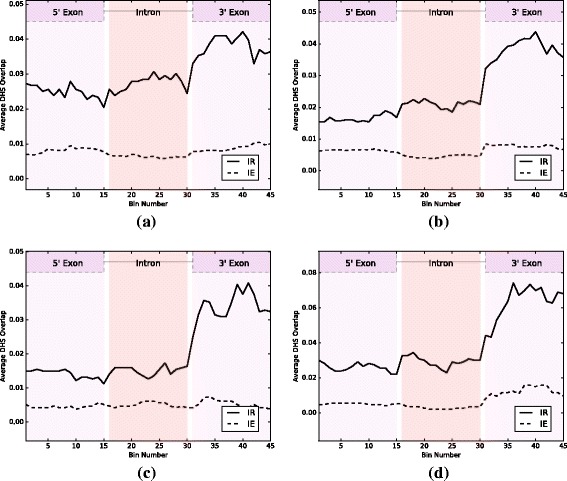
DHS content profiles in IR and IE. For each sequence bin within an IR/IE event we show the frequency with which that bin overlaps a DHS. Profiles are computed for arabidopsis leaf samples (**a**), arabidopsis flower samples (**b**), rice leaf samples (**c**), and rice callus samples (**d**). In all samples, we see overall higher DHS occupancy across IR events compared to IE events, suggesting a more open chromatin in IR. Moreover, the DHS content is much higher in the 3’ exons of IR events

**Fig. 2:**
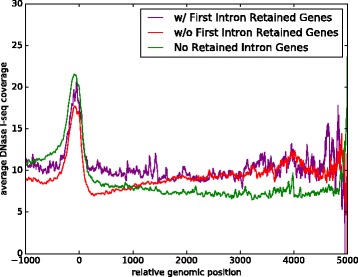
Profile of DNase I-seq read coverage across genes. DNase I-seq read coverage is shown for all genes whose length is up to 5000bp. Genes whose first intron is retained (purple) have higher overall coverage close to the TSS (coordinate 0 on the x-axis). Genes with no IR (green) have lower overall coverage than genes that exhibit IR anywhere except the first intron (red) or those that exhibit IR in the first intron (purple). Note that the profile extends 1000bp upstream of the TSS with a clear peak in the promoter region

Our first goal is to investigate the relationship between IR and chromatin accessibility. For this task we analyzed existing DNase I-seq data in both arabidopsis and rice for which RNA-seq data for the same samples is also available [26, 27]. First, we used the RNA-seq data to identify events where an intron is retained (IR), and events where there is no evidence for IR, which we refer to as intron excision (IE). Note that we do not use the term “constitutive splicing”, as other alternative splicing events could be occurring. The DNase I-seq data associated with those samples were then used to identify peaks representing DHSs. We observe that IR events tend to overlap DHSs to a much greater degree than IE events: 13.3- 26.5% of IR events overlap a DHS compared to 2.1- 5.2% for IE, a difference that is highly statistically significant (see Table 1, Fig. 1, and Additional file 1: Table S2 and S4 for details). Since expressed genes typically exhibit a large peak in DNAse I-seq coverage in their promoter region (see Fig. 2), we excluded IR/IE events in the first intron of a gene. Consistent with the above results and the higher chromatin accessibility of the first introns, they exhibit significantly higher rates of IR than other introns in both arabidopsis and rice with a *p*-value of 5.90×10^−89^ in arabidopsis and a *p*-value of 8.93×10^−25^ in rice using the Fisher exact test.

**Table 1:** Enrichment of DHSs in IR and IE events

Data Source	DHS Content		*p*-value
	IR	IE	
Arabidopsis (leaf) [26]	15.24%	4.02%	1.07×10^−66^
Arabidopsis (flower) [26]	13.28%	3.49%	9.43×10^−93^
Rice (leaf) [27]	16.07%	2.13%	2.29×10^−123^
Rice (callus) [27]	26.46%	5.21%	3.61×10^−104^

DHS content is the fraction of IR/IE events with an overlapping hypersensitive site. The significance of the difference is quantified by the Fisher exact test

### IR events exhibit higher chromatin accessibility than IE events

**Fig. 3:**
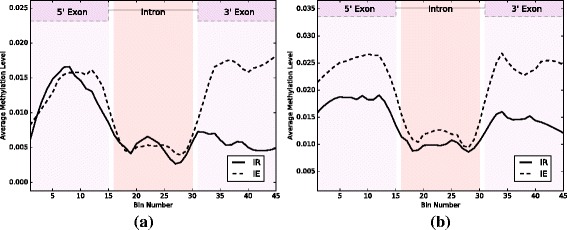
Methylation profiles in IR and IE Methylation levels are shown across introns and their flanking exons in IR and IE events in arabidopsis (**a**) and rice (**b**)

As a complement to the analysis of DHSs detected using peak calling, we compared IR and IE events on the basis of raw DNase I-seq read depth (see Additional file 1: Figure S1). In agreement with the higher proportion of DHSs associated with IR, we observe that IR events have a much higher mean DNase I-seq coverage than IE events (*p*-value of 1.22×10^−56^ in arabidopsis, and a *p*-value of 5.25×10^−100^ in rice using the Mann–Whitney U test [28]), demonstrating that chromatin is more open in IR events than in IE events. As further evidence we analyzed methylation profiling data in arabidopsis and rice, and found that IR events exhibit lower methylation levels in the 3’ exon (see Fig. 3). This is consistent with the results we reported using DNase I-seq data, as DNA methylation has been reported to have an inverse correlation with chromatin accessibility [29].

### Protein footprint analysis

**Fig. 4:**
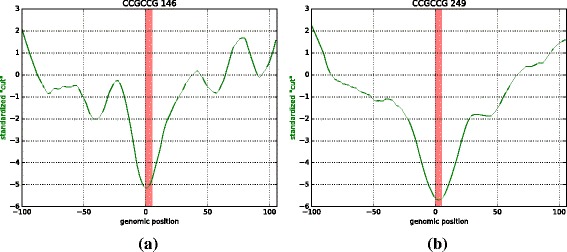
HMM footprint detection. The hexamer CCGCCG was detected to have a footprint at the location of the hexamer (red bar) in the standardized DNase I-seq data profile in both arabidopsis (**a**) and rice (**b**). The number of occurrences of the hexamer in IR/IE events is shown next to the k-mer in the title of each sub-figure. The profile extends 100bp upstream and downstream of the hexamer location, and is used by our HMM to score the k-mer for a potential footprint. In both cases, we see a clear dip in coverage indicating a possible footprint at the hexamer location

Previous studies have used DNase I-seq data to detect potential transcription factor binding sites in promoter regions by searching for a dip in the DNase I-seq coverage [22]: a region of more accessible chromatin is interpreted as the “footprint” left by protein binding. Since splicing occurs co-transcriptionally, there is a potential for events at the DNA level to directly affect splicing, e.g. via recruitment of splicing factors through their interaction with DNA-binding proteins [17]. We used a continuous Hidden Markov Model (HMM) described in the Methods section to discover the footprints of protein binding by searching for a footprint in all occurrences of a given hexamer. A representative footprint is shown in Fig. 4, which shows the DNase I-seq data profile for the hexamer CCGCCG, that was detected by our HMM to have a footprint in 3’ exons, in both arabidopsis and rice. This hexamer is over-represented in IR events (*p*-value of 0.0008 in arabidopsis, and a *p*-value of 1.07×10^−24^ in rice, computed using the Fisher exact test).

We performed a comprehensive analysis across all hexamers to detect those that have a footprint and exhibit an association with IR or IE in the arabidopsis and rice leaf data. Our first observation is that in IR events the majority of the hexamers come from the 3’ exon, while for IE, all the hexamers are intronic (see Table 2 for details). In rice we identified a much larger number of hexamers in IR events, likely due to greater read coverage of the DNase I-seq data (see Additional file 1: Table S1). A complete list of the hexamers that were detected is provided in the Additional file 2.

**Table 2:** Enriched hexamers exhibiting a footprint

Sample	IR Events			IE Events		
	5’ Exon	Intron	3’ Exon	5’ Exon	Intron	3’ Exon
Arabidopsis (leaf) [26]	12	6	100	0	28	0
Arabidopsis (flower) [26]	4	3	105	0	27	0
Rice (leaf) [27]	88	75	262	0	14	0
Rice (callus) [27]	46	32	192	0	30	0

For each of the four datasets we provide the number of hexamers that exhibit a footprint and are also enriched in either IR or IE events. The number of enriched footprint-hexamers are shown in each of the three regions of an event: 5’ exon, intron and 3’ exon. An HMM score cutoff of *S*=0.30 was used to generate the footprint hexamers

Many of the hexamers we identified are conserved in arabidopsis and rice: In the 3’ exon 246 hexamers were common between the two species, while 19 are conserved in the intronic region of IE events. This level of overlap is highly statistically significant (*p*-values of 2.25×10^−165^ and 2.10×10^−32^ respectively, in a hypergeometric test). This level of conservation is strong support for the functional importance of these hexamers. We note that for finding conserved hexamers we used a looser threshold for footprint calling, as the requirement of conservation provided an additional level of filtering of potential false positives. Manual inspection of the detected hexamers showed that all of them exhibited valid footprints.

**Fig. 5:**
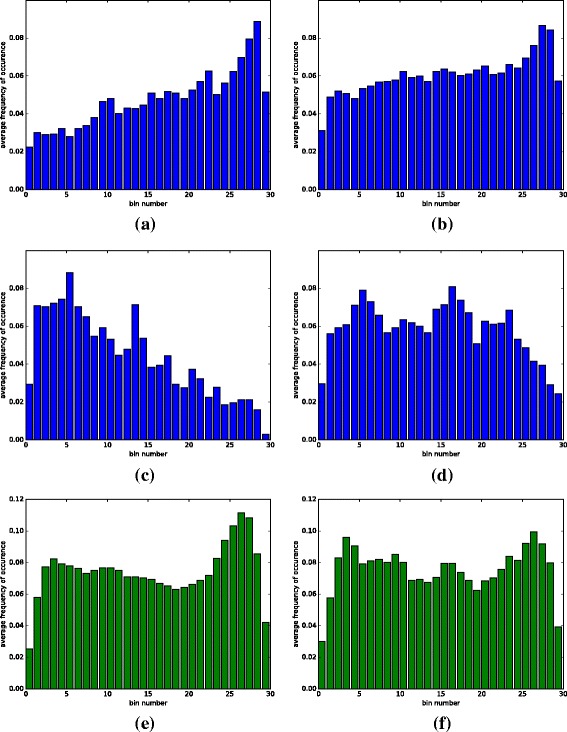
Hexamer positional preference. Average positional preference is shown for AT-rich footprint-exhibiting hexamers in the 3’ exon region of IR events (**a**), and for comparison, the same hexamers in the 3’ exon region of IE events are shown (**b**). Similarly, (**c**) and (**d**) show average positional preference for GC-rich hexamers in the 3’ exon region of IR and IE events, respectively. To demonstrate the positional preference of footprint-exhibiting hexamers that are associated with IE events we show the average positional profile of those hexamers in IE events (**e**) and IR events(**f**)

The conserved hexamers in leaf tissue were clustered into motifs that are summarized in Table 3; their motif logos can be found in Additional file 1: Table S7. In IE we detected motifs only in the intron; these motifs are T-rich with a few As and no Gs or Cs. The converse holds for intronic motifs in IR: they are GC rich with few As and no Ts. Furthermore, occurrences of the intronic IE motifs show a clear pattern in terms of their preferred position within the intron, with a very clear peak near the 3’ of the intron, and are likely associated with the polypyrimidine tract (see Fig. 5). No such pattern is observed for the IR intronic motifs.

**Table 3:** Common enriched footprint-hexamers between arabidopsis and rice

Event type	Region	hexamers	*p*-value	Motif consensus
IR	5’ Exon	13	1.70×10^−07^	CGCCG,(G/C)(G/C)GCGG,(A/G)T(C/T)(G/T)(C/G)A
	Intron	2	0.27	AAGGAG,CGGCGG
	3’ Exon	246	2.25×10^−165^	AAAA,AAATT,CCGAC,CGCxCG,(C/A)TTT, GCGGC,GxTTT,(T/G)AAA,TTT(C/T) (G/T)T(C/T)(C/G)(G/A)
IE	5’ Exon	0	N/A	-
	Intron	19	2.10×10^−32^	TTAA(T/A)(T/A),T(T/A)TTT(A/T)
	3’ Exon	0	N/A	-

The number of hexamers in common between the arabidiopsis and rice leaf samples, and the corresponding significance levels of the overlap are shown for all three regions of IR and IE events. The hexamers in each region were clustered, and motif consensus sequences are shown. When there is no clear consensus in a given position, that is denoted by an x. Leading or trailing positions without a clear consensus were omitted, so some consensus sequences are less than 6 nucleotides long. In the intronic region of IR events only 2 hexamers were detected so no clustering was performed. Here, an HMM score cutoff of *S*=0.20 was used with manual verification of the footprints of the overlapping hexamers

Most of the hexamers and motifs associated with IR events occur in the 3’ exon; the majority of them (6/10) are AT-rich, and some of the rest (3/10) are GC-rich. Both sets of motifs exhibit very different positional preferences: the AT-rich motifs tend to occur at the 3’ end of the exon, while the GC-rich motifs tend to occur in the 5’ end of the exon (see Fig. 5 for the overall positional preferences of those motifs, and Additional file 1: Figure S4 for positional preferences of individual hexamers). We believe that the positional preferences observed reflect different biological roles of these motifs in regulating IR and IE events, as discussed below.

In order to find potential proteins associated with our hexamers we searched the all the arabidopsis hexamers against a collection of 410 transcription factor motifs from the Plant Cistrome Database [30] as described in the Methods section. Out of 280 enriched hexamers, 96 of them had at least one match. The breakdown into the different locations is found in Additional file 1: Table S8. The matching motifs come from a variety of families of transcription factors. The largest number of matches was to the AP2/EREBP family, which is a plant-specific family of DNA-binding proteins [31]. The second-largest number of matches were to Dof proteins through hexamers in the 3’ exon that contain mostly A or T nucleotides; this family of transcription factors is also plant-specific [32]. C2H2 DNA-binding proteins are also strongly represented. Interestingly, a vast majority (about 60%) of them have been shown to be involved in the regulation of AS in animals [33], although the effect could be either direct or indirect, through the regulation of splicing regulators. Some of these effects are likely to be direct since DNA-binding proteins, including transription factors, have been shown to bind in gene bodies [34]. Complete details of the matches are found in Additional file 3. These results implicate plant transcription factors in splicing regulation. This is in agreement with recent results in mammals that revealed that more than a third of splicing regulators detected in a high-throughput screen were transcription factors [33].

**Fig. 6:**
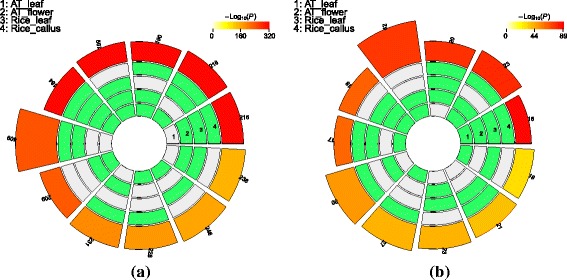
Significance of overlap of enriched hexamers. The significance of overlap among enriched hexamers exhibiting a footprint is shown in the 3’ exon region of IR events (**a**), and in the intronic region of IE events (**b**) for two or more samples. The overlap is shown in circular layout for all possible combinations of two or more of the four samples. The four inner sections of each slice represent the four samples and a sample is labeled in green if it is included in a particular combination. The right most slide provides the labeling of the samples. The size of the fifth section in each slice represents the number of hexamers in an intersection of the corresponding samples. The actual number of overlapping hexamers is also shown. Finally, the color of the fifth section indicates the significance of overlap (*p*-value). The intersections are sorted based on *p*-value, starting at the labelled segment in an anti-clockwise fashion

Next, we performed an additional enrichment analysis to test the significance of the overlap across all four datasets (arabidopsis leaf and flower tissue and rice leaf and callus). We used the *SuperExactTest* [35] to quantify the overlap between all subsets of samples simultaneously. Since the majority of hexamers occurred in the 3’ exon of IR events and intronic part of IE events, we performed this analysis in those regions. The results shown in Fig. 6 demonstrate a large and highly statistically significant overlap even when considering all combinations of samples.

## Discussion

Splicing occurs co-transcriptionally, and there is increasing evidence indicating that chromatin organization involving epigenetic marks and rate of transcription regulate alternative splicing in mammalian systems [17]. However, in plants, virtually nothing is known in terms cotranscriptional regulation of alternative splicing. Here we investigate the role of chromatin architecture and potential DNA elements that may regulate IR.

In our data we observe a greater number of DHSs in IR events compared to IE events, and this is most prominent in the 3’ exon. A similar pattern was observed in the raw DNase I-seq data as well. We present two possible hypotheses by which this increase in open chromatin contributes to IR. Splicing is a much slower process than transcription [17], and we hypothesize that the less open chromatin in IE events leads to more PolII pausing (the speed-bump model), which allows for a greater degree of recruiting of splicing factors and hence greater likelihood of intron recognition. Conversely, in retained introns, because of the higher elongation rates, there is less chance of recognizing the splice sites, leading to IR. The fact that retained introns have weaker splice sites [13, 36], makes them more sensitive to the rate of elongation. However, this hypothesis does not take into account that the increased prevalence of DHSs could be due to binding of *trans*-factors, and also does not account for the much larger number of hexamers with footprints that are associated with IR. For example, in the arabidopsis leaf data we found 118 hexamers with footprints that are enriched in IR, and only 28 in IE.

The increased number of footprints that we observed in IR could be the result of one of two factors: 1. Increased PolII pausing and/or, 2. Binding of other chromatin/DNA-interacting proteins. Braunschweig et al. have recently shown that in mammalian systems retained introns are associated with increased PolII pausing [13]. This pausing may lead to recruitment of splicing suppressors that compete or prevent splicing activators from binding, leading to IR. There is data supporting this hypothesis in non-plant systems [13], and this hypothesis is consistent with the observation that the high rate of DHS occurrence in the 3’ exon is coupled with the occurrence of a much higher number of hexamers with footprints that are associated with IR. This suggests a key role for chromatin architecture in the 3’ exon in regulating the splicing of the upstream intron. We believe the second mechanism is more likely; however additional work aimed at assaying PollII occupancy in retained vs excised introns is required to help distinguish between these two mechanisms.

Chromatin modifications have recently been associated with IR in humans: Braunschweig et al. have shown that the chromatin activation mark H3K27ac is enriched in retained introns [13]. This observation is consistent with our result showing greater DHS frequency in retained introns: this modification is associated with more flexible chromatin structure, which facilitates the interaction of proteins with IR regulatory elements.

The AT-rich hexamers in IE have a positional preference for the 3’ end of the intron, which suggests they are likely associated with the polypyrimidine tract, which in plants is T-rich [1], leading to more efficient recognition of splice sites. In contrast, the hexamers we detected in the introns of IR events, show very different base composition, with virtually no Ts, likely resulting in poor recognition of these introns.

DNA methylation has been shown to regulate alternative splicing, including IR, in plants and animals [37–40]. Part of this regulation could be due to reorganization of chromatin; in support of this, it has been shown that there is an inverse relationship between DNA methylation and open chromatin [29]. In our analysis we found a strong correlation between open chromatin and reduced methylation in IR vs IE events in both arabidopsis and rice. Open chromatin may make the DNA more available to binding by DNA-binding proteins. In our hexamer analysis we found that the majority of those hexamers occur in the 3’ flanking exon, which demonstrated the highest level of open chromatin. Interestingly, the motifs in the introns of IR events are either CG- or AG-rich. Hence, it’s possible that the hexamers enriched in CG di-nucleotides are the targets of methylation, which in turn could attract splicing suppressors, either directly, or through methylation-binding proteins [37]. Alternatively, proteins bound to methylated regions can modulate the rate of elongation of PolII [37, 41]. Further studies are required in order to confirm or exclude some of these possibilities.

In addition to the matches in the Plant Cistrome Database described above, we identified other transcription factors that have DNA binding motifs that match the hexamers discovered by our pipeline. These include Homeodomain-leucine zipper (HD-Zip) proteins, which are a family of transcription factors unique to plants [42] have DNA binding sequences that match some of the AT-rich hexamers that were detected in our analysis. For example, *ATHB9*, which is an HD-Zip class II protein, was shown to have affinity for the sequence GTAAT(G/C)ATTAC; the core AAT(G/C)A segment of this sequence matches multiple conserved hexamers detected in the 3’ exon of retained introns. HD-Zip class IV proteins bind sequences containing a TAAA core, which is consistent with a large number of hexamers both in IR and IE events.

Although epigenetic changes, including DNA methylation and histone modifications have been shown to be important regulators of AS in animals [37, 43, 44], relatively little is known about their role in AS in plants. This work strongly indicates a role for chromatin organization and DNA methylation in IR. Recently Pajoro et al. [45] have shown that histone modifications alter AS in plants, supporting our conclusion that chromatin state is a critical regulator of AS.

## Conclusions

In this work we established a clear correlation between IR and chromatin accessbility and DNA methylation in arabidopsis and rice. We found that chromatin is more open in retained introns, which can be explained using a kinetic model of the splicing process. The observed open chromatin in IR is consistent with the reduced methylation levels we observed in these regions. The more open chromatin in IR also suggests that IR is more highly regulated than constitutive splicing, which is supported by the large number of conserved sequence elements that were discovered in footprints associated with IR. A majority of the discovered sequence elements occur in exons immediately downstream of retained introns, indicating its importance in regulating IR events. Further experiments are required in order to establish the biological function of these sequence elements and to experimentally verify the hypothesized connections between intron retention and chromatin organization.

## Methods

### Data collection

For arabidopsis, read data from Zhang et al. [26] were downloaded from the Gene Expression Omnibus (GEO); data with GEO accession number *GSE*53322 was used. For rice, we used data from Wu et al. [27] (GEO accession number *GSE*26610); The corresponding RNA-seq was published elsewhere [46] (GEO accession number *GSE*33265). For rice, there were two samples coming from two tissues: leaf and callus. For bisulfite-seq, we used raw data from Zemach et al. [47] (GEO accession number *GSE*41302) and Chodavarapu et al. [48] (GEO accession number *GSE*38480), for arabidopsis and rice, respectively.

### Alignment and processing

In case of data from Zhang et al. [26], we used their aligned DNase I-seq and RNA-seq files. For the rest of the data, the raw reads were first pre-processed using *FastQC* [49] and trimmed using *fastx-trimmer* [50] when required. Next, the processed reads were aligned to the corresponding reference genomes (*TAIR10* for arabidopsis and *MSU v7* for rice) using different alignment tools. All the RNA-seq samples were aligned using *Tophat2* [51] with default parameters. The *Tophat2* alignments were filtered to obtain only uniquely aligned reads. The arabidiopsis DNase I-seq data was aligned using *Bowtie* [52]. *Bowtie* was used with the command-line argument -m 1 to suppress multiple alignments. For the rice DNase I-seq data, we used *STAR* [53] to align the reads with the parameters outFilterMultimapNmax 1 and alignIntronMax 1 to adjust for genomic data alignment. The bisulfite-seq data was quality- and adapter-trimmed using *Trim Galore!* [54]. For alignment and methylation calling, we used *bismark* [55]. Note that biological and technical replicates—if there were any—were pooled together for each sample. The alignment statistics are summarized in Additional file 1: Table S1.

### Extraction of IR/IE events and peak calling

To extract IR and IE events we used annotated IR events from the gene models as well as evidence from the RNA-seq data found using SpliceGrapher [56], which is a tool that combines gene models and RNA-seq data to predict alternative splicing events. To avoid any ambiguity between IR and IE events, we used strict criteria to distinguish between the two on the basis of the RNA-seq data: exonic read depth of at least 20 was required for a gene to be considered in our analysis; full coverage across an intron was required for it to be considered retained, and no coverage for it to be considered an intron excision event. The choice of the exonic read depth threshold had little effect on our results (see Additional file 1: Table S4). For DHS peak calling in the DNase I-seq data, in both arabidopsis and rice, we used the Hotspot [57] program with default parameters. Additional file 1: Table S2 summarizes the DHS peaks and the numbers of IR/IE events are provided in Additional file 1: Table S3. When computing the DHS content profile and DNase I-seq coverage profiles across IR/IE events we excluded events involving the first intron of a gene, since the first intron often overlaps the DHS associated with the promoter region, and tends to exhibit higher DNase I-seq coverage than introns further downstream. As a further step for addressing the non-uniformity of DNase I-seq coverage across a gene, for each IR event, we selected IE events with similar relative positions within their genes.

### Protein footprint analysis

#### Hexamer data generation

For the discovery of k-mers that exhibit footprints we chose to focus on hexamers since this provides a good balance of specificity and tractability of exhaustive search. We considered all possible hexamers coming from the three parts of an event: 5’ exon, intron, and 3’ exon. For every hexamer, we generated the DNase I-seq profile. For each occurrence of the hexamer we extracted its DNase I cut at every nucleotide position of the hexamer as well as 100bp upstream and downstream of its location and then took the average over all positions. Note that in going 100bp upstream and downstream, we made sure not to go beyond the boundaries of the event parts: intron or the flanking exons. This was done to avoid introducing any bias coming from the properties of different segments of the event. In case of multiple instances of a hexamer in a sequence, we considered the one which had the lowest DNase I-seq coverage.

#### Footprint calling using continuous HMMs

**Fig. 7:**
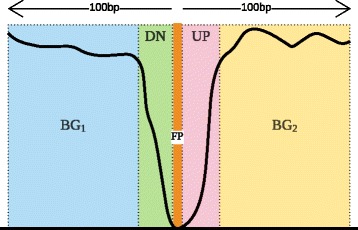
HMM Architecture The core continuous HMM states used to discover footprints are shown. The five states represent different regions of the DNase I-seq coverage profile: leading background (*BG*_1_), down (*DN*), footprint (*FP*), Up (*UP*), and trailing background (*BG*_2_). The footprint state is shown in the center, within the “dip” in the DNase I-seq coverage

We used the profile of DNase I-seq coverage to call footprints using a continuous HMM. Continuous HMMs are a good modeling tool for sequences of real values such as DNase I-seq coverage, and allow us to detect whether the observed profile contains a feature that can be identified as a protein footprint. Our model was inspired by a similar model [58] and the implementation uses SageMath [59]. As shown in Fig. 7, our HMM has five core states: the leading background state (*BG*_1_), the down state (*DN*), the footprint state (*FP*), the up state (*UP*) and finally, the trailing background state (*BG*_2_). The HMM was trained on data profiles of hexamers with manually verified footprints and was used to score the rest of the hexamers. Note that all hexamer profiles were standardized to a background score calculated from the training set. To account for tandem motifs, we added additional states to the model to represent secondary footprints upstream or downstream of the primary footprint. The state diagram for the final HMM, which has 13 states, along with complete specification of the model (transition and emission probabilities), and the training and testing protocol, can be found in the Additional file 1: Tables S5 and S6, and Figure S3.

Using the trained HMM we score hexamers as potential footprints using the following expression:

$$S = -\log \biggl[\frac{C_{\mathit{FP}}}{C_{\mathit{BG}}} \biggr], $$

where *C*_*FP*_ is the average standardized coverage at the footprint state and *C*_*BG*_ is the average coverage across the background states. A conservative threshold of *S*=0.30 was used in the analysis of individual hexamers, and the cutoff was lowered to *S*=0.20 in the cross-species analysis. To cluster the hexamers into motifs, we used complete linkage hierarchical clustering with a distance metric that assigns two k-mers a distance of 0 if they share a 4-mer; their edit distance is used otherwise; clusters were cut at a depth of 4. We used *clustalw2* [60] to generate the multiple alignments which were then fed to *weblogo* [61] to generate motif logos. For positional preferences, when a hexamer occurred multiple times in an IR/IE event, we chose the one with lowest DNase I-seq read depth among all occurrences.

#### Motif matches in the plant cistrome database

All significantly enriched arabidopsis hexamers were searched against each motif from the Plant Cistrome Database [30] using their respective position weight matrices. A cistrome motif was considered a match for a given hexamer if the hexamer matched exactly the consensus sequence at some location, such that the information content in the positions covered by the hexamer consist of at least 50% of the overall information content of the motif.

### Statistical tests

Whenever testing multiple hypotheses, the resulting *p*-values were adjusted using the Benjamini-Hochberg method [62]. All the statistical tests used in this work were performed in R; for the significance of multi-sample intersections, we used the R package for the super-exact test [35] with population size of 4096.

## Additional files

Additional file 1 SupplementaryFile_1.docx. Additional figures and tables are provided in this file. (DOCX 2507 kb)

Additional file 2 SupplementaryFile_2.xlsx. This file lists footprint-exhibiting hexamers for all four individual samples in separate tabs for IR and IE events. In addition, the footprint-exhibiting hexamers common between the arabidopsis and rice leaf samples are provided in the last two tabs for IR and IE events, respectively. (XLSX 78 kb)

Additional file 3 SupplementaryFile_3.xlsx. This file provides a list of the footprint-exhibiting hexamers in arabidopsis that match motifs in the Plant Cistrom Database. (XLSX 64 kb)

## Abbreviations

AS: alternative splicing
DHS: DNase I hypersensitive site
DNase I: Deoxyribonuclease I
GEO: Gene Expression Omnibus
HMM: hidden Markov model
HD-Zip: Homeodomain-leucine zipper
IR: intron retention
IE: intron excision
PolII: RNA polymerase II
SRE: splicing regulatory element
