# Supplementary material for: Exploring the relationship between intron retention and chromatin accessibility in plants
Source: BMC Genomics. 2018 Jan 5;19:21. doi: 10.1186/s12864-017-4393-z (PMC5756433; doi:10.1186/s12864-017-4393-z)
Supplement: Supplementary file 1 — SupplementaryFile_1.docx. Additional figures and tables are provided in this file. (DOCX 2507 kb) [file 12864_2017_4393_MOESM1_ESM.docx]

**Supplementary Material**

**Exploring the relationship between intron retention and DNase I hypersensitivity in plants**


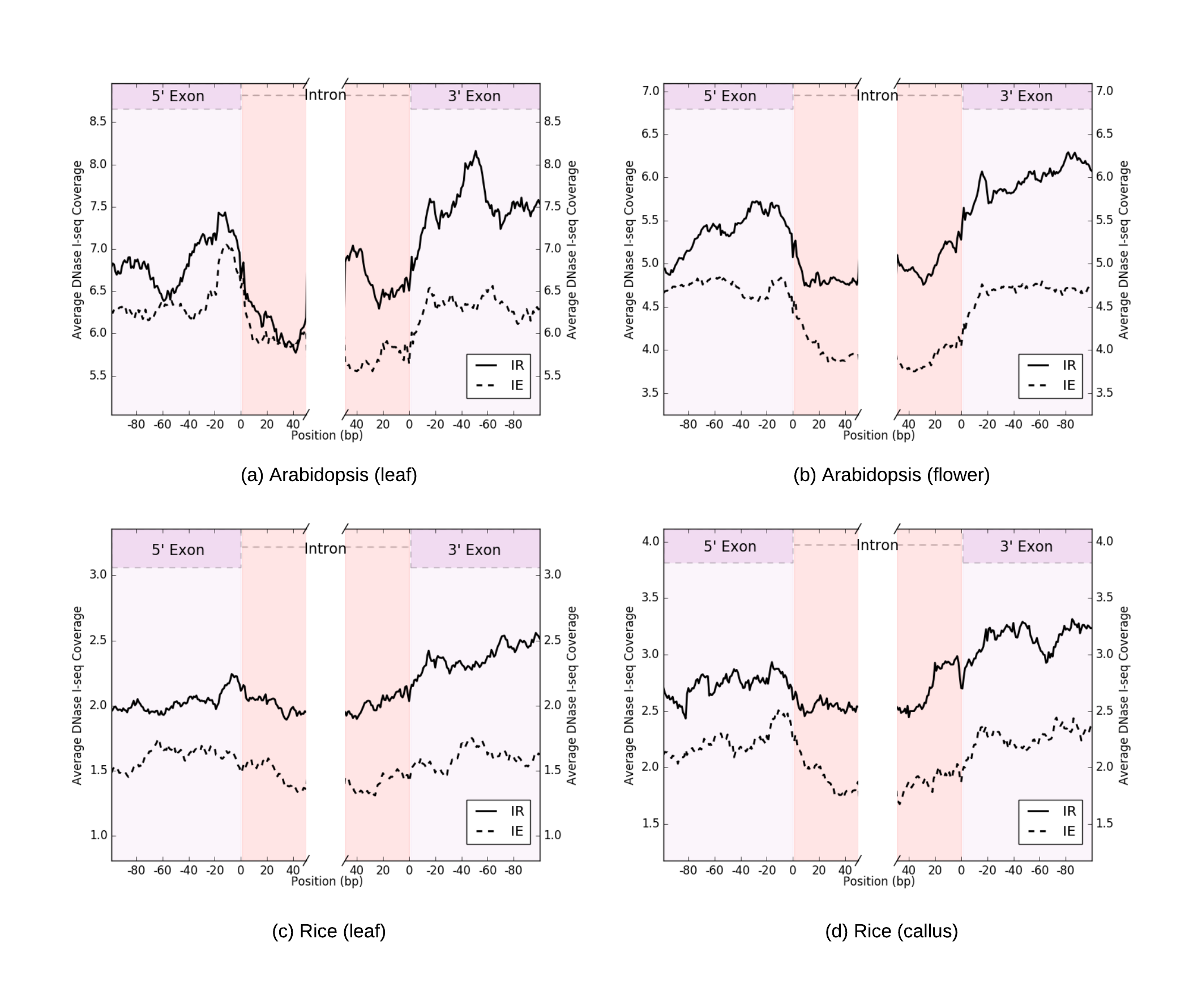


**Figure 1 Average DNase I-seq coverage profile across IR and IE events**

Average DNase I-seq coverage profile is shown across IR and IE events in the four samples: Arabidopsis leaf (a) and flower (b); rice leaf (c) and callus (d). The profile is centered at the 5’ and 3’ splice sites (indicated by “0” on x-axis in the split figure), and goes 50bp into the intron and 100bp into the flanking exons. Note that we chose all three parts of an IR/IE event to be at least 100bp. These profiles do not include events that come from the first intron of a gene. Moreover, to avoid bias, for each IR event, we selected IE events with similar relative positions within the gene.


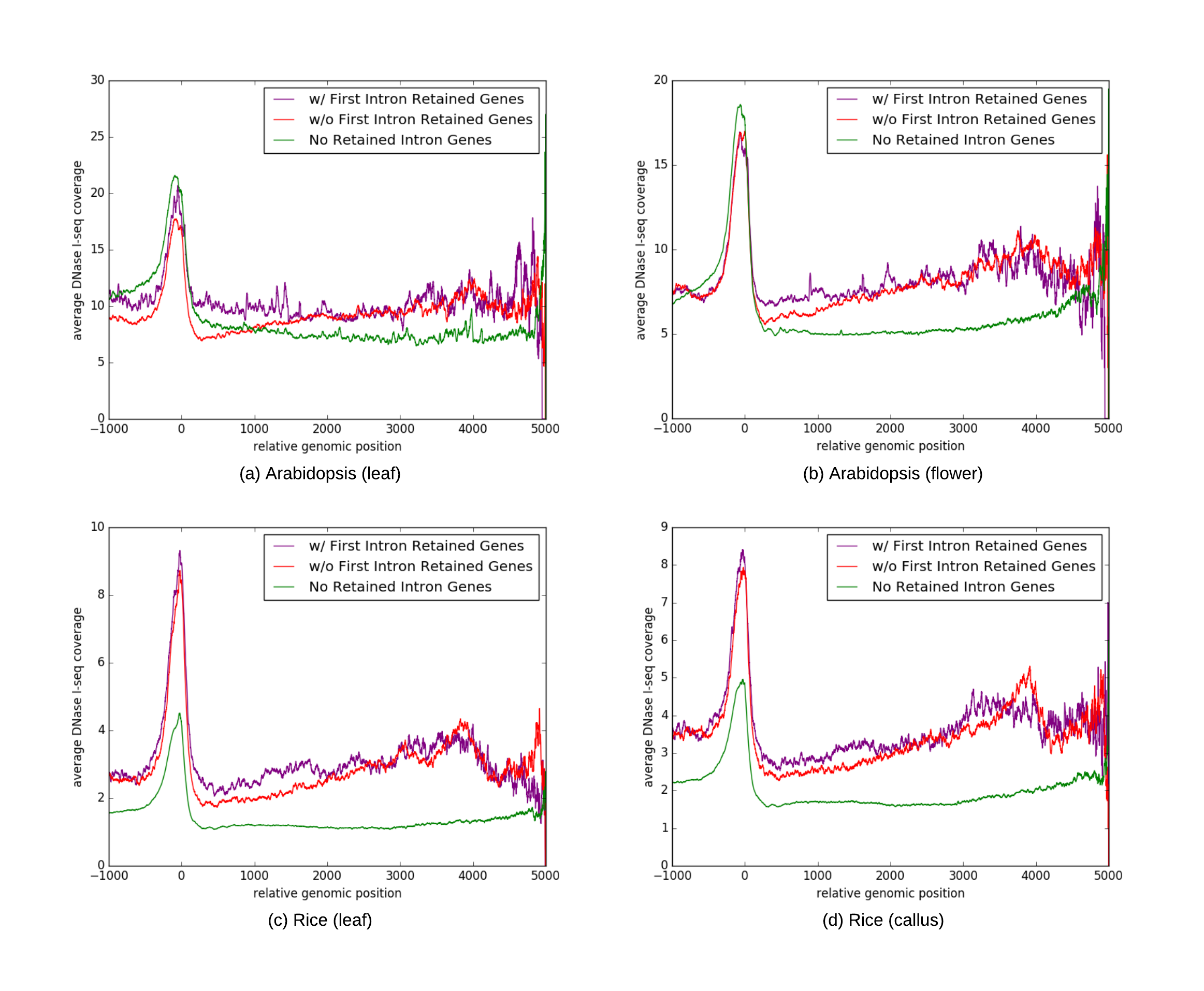


**Figure 2 Average DNase I-seq coverage profile across genes that are up to 5000bp in length.**

Average DNase I-seq coverage profile for the four samples: Arabidopsis leaf (a) and flower (b); rice leaf (c) and callus (d). In each case, a pool of genes up to 5000bp in length are used (roughly 95% of total genes). The profile encompasses the gene body and 1000bp upstream of the transcription start site represented by ‘0’ on the x-axis. Each figure shows the profile for three sub-categories: genes with first intron retained (purple), genes with intron(s) retained anywhere else but the first one (red), and genes without any retained intron (green).

**
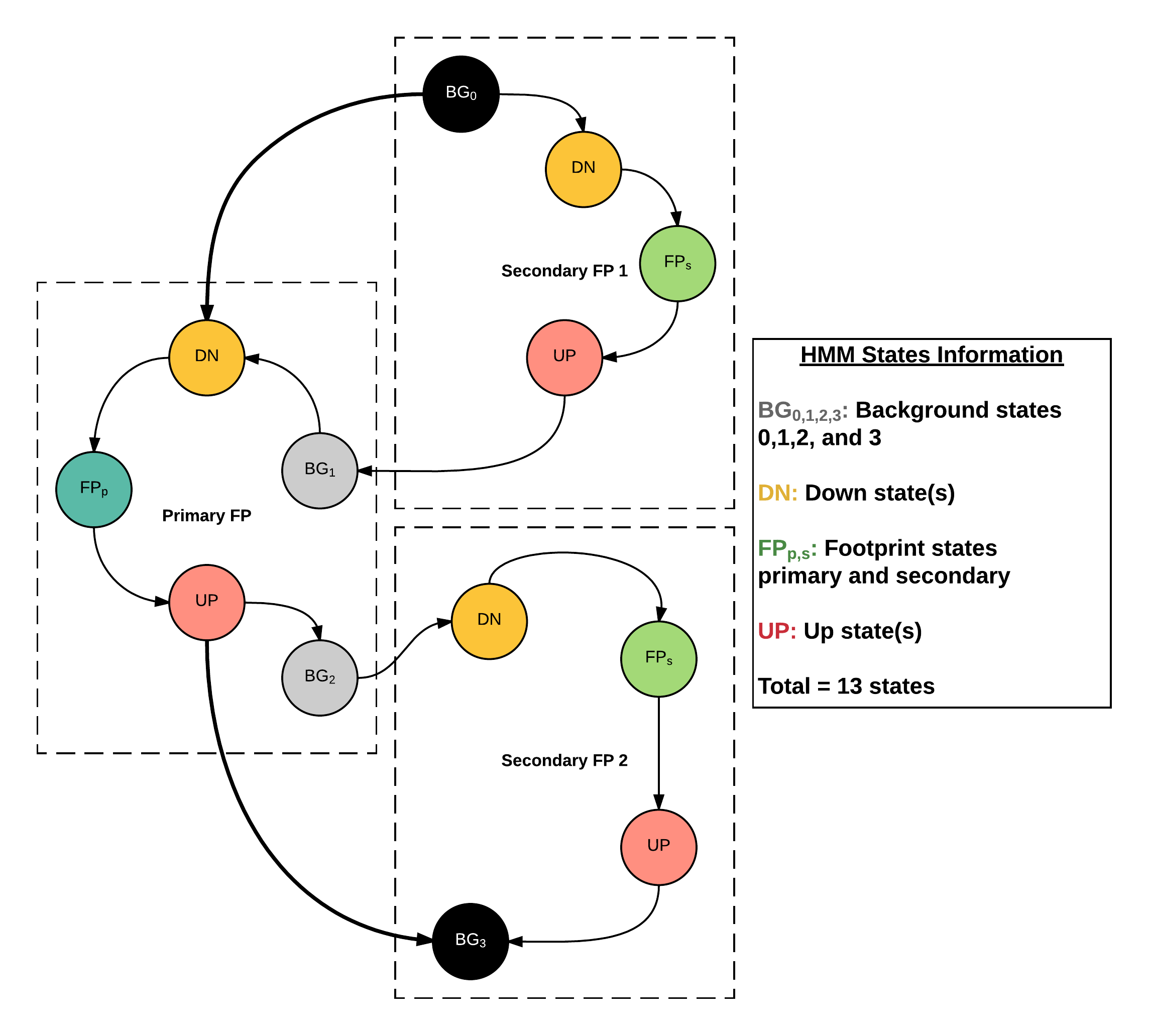
**

**Figure 3 Continuous HMM state diagram**

The complete state diagram for the continuous HMM used to predict hexamers with potential footprints. The diagram shows all 13 states. The HMM consists of three modules, to enable us to model leading/trailing footprints in addition to the primary footprint. Each module has copies of the five core states. The size of the arrow (transition) as in BG_0_→DN and UP→BG_3_ represents higher probabilities than the other transitions from the same state. These probabilities are highlighted in the supplementary table 4. This is used to emphasize the primary footprint detection by our model in all cases. The figure also summarizes the HMM states in the rectangular box to the right.

**
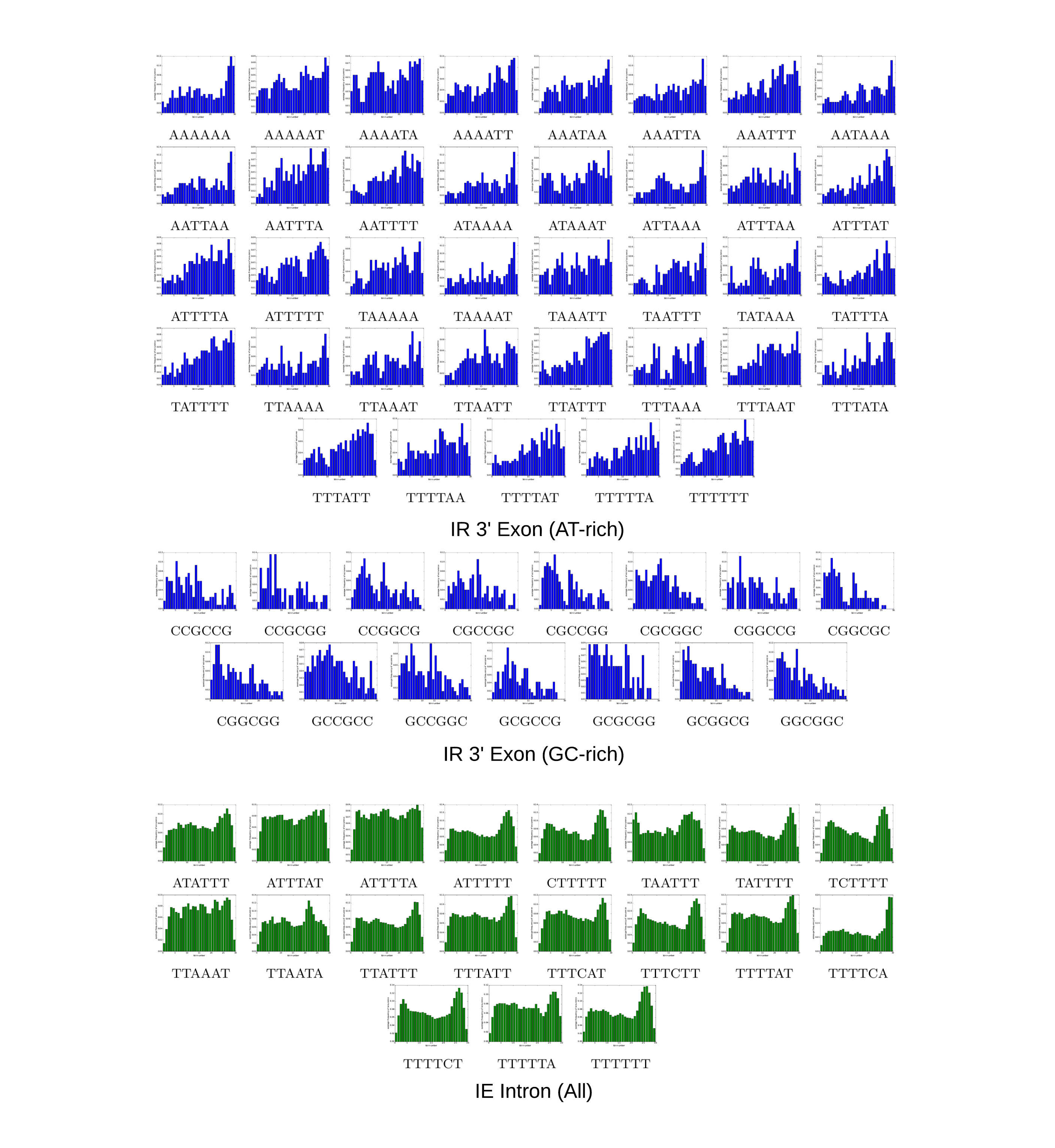
**

**Figure 4 Positional preferences of hexamers**

Positional preference is shown for AT-rich hexamers (top), GC-rich hexamers (middle) in 3’ exon region of IR events, and all hexamers in intron region of IE events (bottom). All hexamers mentioned in the figure exhibit a footprint.

**Table 1 Alignment statistics**

Alignment statistics for different *Arabidopsis* *thaliana* (AT) and rice samples. Note that the aligned reads went through preprocessing and then aligned using tophat2 for RNAseq and bowtie/STAR for DNase I-seq (see Methods in the main text). The reads in both cases (DNase I-seq and RNA-seq) were filtered for multiple alignments and filtered for spurious junctions for the RNAseq. Also, in all samples, biological and technical replicates were pooled. As mentioned in the main text, we used pre-aligned DNase I-seq and RNA-seq from [1].

| **Type of data** | **Sample** | **Total Reads** | **Aligned reads (Unique/Filtered)** |
| --- | --- | --- | --- |
| DNase I-seq | Rice (leaf, control) [2] | 42593905 | 29260669 (68.70%) |
|  | Rice (callus, control) [2] | 57037438 | 39867789 (69.90%) |
| RNA-seq | Rice (leaf, control) [2] | 40206025 | 37364769 (92.93%) |
|  | Rice (callus, control) [2] | 29634838 | 27100117 (91.45%) |
| Bisulfite-seq | Arabidopsis [3] | 41177470 | 16559509 (40.20%) |
|  | Rice [4] | 130128482 | 62386292 (47.90%) |

**Table 2 Peak calling statistics (DHSs)**

Hotspot was used to call DHS peaks in all DNase I-seq samples. Rice samples, on average, had more DHS peaks identified. Since hotspot can’t handle replicates, we pooled DNase I-seq libraries.

| **S.No.** | **Sample** | **# of DHSs** |
| --- | --- | --- |
| 2 | AT (leaf) [1] | 45,665 |
| 3 | AT (Flower) [1] | 42,782 |
| 4 | Rice (Leaf) [2] | 69,277 |
| 5 | Rice (Callus) [2] | 107,092 |

**Table 3 Summary of IR and IE events**

Number of IR and IE events extracted at different coverage levels are listed below. Evidence from known gene models and RNA-seq data was used to extract the events as described in the Methods section in the main paper.

| **Sample** | **Expression Level** | **IR Events** | **IE Events** |
| --- | --- | --- | --- |
| ***AT (Leaf) [1]*** | 1 | 3804 | 63538 |
|  | 5 | 3599 | 50666 |
|  | 10 | 3196 | 38568 |
|  | 20 | 2397 | 21426 |
| ***AT (Flower) [1]*** | 1 | 5007 | 64005 |
|  | 5 | 4856 | 54665 |
|  | 10 | 4568 | 47229 |
|  | 20 | 3811 | 33936 |
| ***Rice (Leaf) [2]*** | 1 | 3882 | 33945 |
|  | 5 | 3579 | 24089 |
|  | 10 | 3254 | 17850 |
|  | 20 | 2619 | 10522 |
| ***Rice (Callus) [2]*** | 1 | 2758 | 40757 |
|  | 5 | 2426 | 30514 |
|  | 10 | 1980 | 23189 |
|  | 20 | 1399 | 13446 |

**Table 4 DHS occupancy in IR and IE events**

| **Sample** | **Expression Level** | **IR Events** | | | | | **IE Events** | | | | | **Fisher Pval** | **IR (%)** | **IE (%)** |
| --- | --- | --- | --- | --- | --- | --- | --- | --- | --- | --- | --- | --- | --- | --- |
|  |  | ***5' Exon*** | ***Intron*** | ***3' Exon*** | ***All*** | ***Total*** | ***5' Exon*** | ***Intron*** | ***3' Exon*** | ***All*** | ***Total*** |  |  |  |
| **AT (Leaf) [1]** | 1 | 61 | 61 | 224 | 346 | 2776 | 498 | 614 | 1102 | 2214 | 54475 | 9.55E-69 | 12.46 | 4.06 |
|  | 5 | 59 | 61 | 220 | 340 | 2643 | 384 | 448 | 839 | 1671 | 43755 | 9.57E-76 | 12.86 | 3.82 |
|  | 10 | 56 | 59 | 202 | 317 | 2341 | 288 | 331 | 630 | 1249 | 33383 | 1.92E-76 | 13.54 | 3.74 |
|  | 20 | 45 | 51 | 172 | 268 | 1758 | 173 | 189 | 381 | 743 | 18480 | 1.07E-66 | 15.24 | 4.02 |
| **AT (Flower) [1]** | 1 | 70 | 70 | 308 | 448 | 3701 | 475 | 484 | 1348 | 2307 | 54779 | 1.44E-78 | 12.10 | 4.21 |
|  | 5 | 66 | 69 | 300 | 435 | 3610 | 354 | 363 | 1121 | 1838 | 47376 | 2.41E-84 | 12.05 | 3.88 |
|  | 10 | 63 | 68 | 285 | 416 | 3400 | 282 | 281 | 946 | 1509 | 41185 | 3.36E-88 | 12.24 | 3.66 |
|  | 20 | 56 | 64 | 259 | 379 | 2854 | 192 | 194 | 653 | 1039 | 29745 | 9.43E-93 | 13.28 | 3.49 |
| **Rice (Leaf) [2]** | 1 | 66 | 58 | 308 | 432 | 3130 | 182 | 172 | 413 | 767 | 29434 | 3.00E-143 | 13.80 | 2.61 |
|  | 5 | 62 | 57 | 297 | 416 | 2915 | 114 | 96 | 286 | 496 | 21309 | 6.77E-148 | 14.27 | 2.33 |
|  | 10 | 58 | 57 | 290 | 405 | 2655 | 75 | 66 | 219 | 360 | 15862 | 2.92E-147 | 15.25 | 2.27 |
|  | 20 | 44 | 50 | 248 | 342 | 2128 | 37 | 33 | 130 | 200 | 9369 | 2.29E-123 | 16.07 | 2.13 |
| **Rice (Callus) [2]** | 1 | 67 | 61 | 339 | 467 | 2177 | 404 | 378 | 1404 | 2186 | 35384 | 3.43E-112 | 21.45 | 6.18 |
|  | 5 | 54 | 54 | 319 | 427 | 1950 | 244 | 191 | 1030 | 1465 | 27076 | 1.40E-120 | 21.90 | 5.41 |
|  | 10 | 42 | 45 | 290 | 377 | 1607 | 164 | 120 | 766 | 1050 | 20776 | 4.77E-122 | 23.46 | 5.05 |
|  | 20 | 34 | 40 | 225 | 299 | 1130 | 99 | 68 | 466 | 633 | 12140 | 3.61E-104 | 26.46 | 5.21 |

DHS overlap statistics are shown for the four samples in both IR and IE events at the four levels of read coverage. For both IR and IE events, the number of DHS (peaks) overlapping the events is shown at both individual parts (5’ exon, Intron, and 3’ Exon) and the whole event (shown in the column titled “All”). The total number of events are also shown for both IR and IE. Note that the numbers shown here are different than what we see in Table 3 as the events containing first introns are filtered out. Finally, the fisher exact test p-value is shown for each case, indicating that the overlap is significant in IR events in contrast to IE events. The last two columns show percent DHS content for both IR and IE events.

**Table 5 HMM transition probabilities**

The HMM’s transition probabilities for all 13 states. The probabilities were derived from the training data (8 hexamers that were manually detected to have a footprint). Some of the probabilities were manually tweaked to adjust for the noise in our data. The highlighted probabilities (as described in figure 2) are relatively higher than the other transition from the same state. This is to force our HMM to prioritize detection of the primary footprint.

| **States** | **BG_0_** | **UP** | **FP_s_** | **DN** | **BG_1_** | **UP** | **FP_p_** | **DN** | **BG_2_** | **UP** | **FP_s_** | **DN** | **BG_3_** |
| --- | --- | --- | --- | --- | --- | --- | --- | --- | --- | --- | --- | --- | --- |
| **BG_0_** | 0.996 | 0.000037239 | 0 | 0 | 0 | **0.004** | 0 | 0 | 0 | 0 | 0 | 0 | 0 |
| **UP** | 0 | 0.042 | 0.958 | 0 | 0 | 0 | 0 | 0 | 0 | 0 | 0 | 0 | 0 |
| **FP_s_** | 0 | 0 | 0.879 | 0.121 | 0 | 0 | 0 | 0 | 0 | 0 | 0 | 0 | 0 |
| **DN** | 0 | 0 | 0 | 0.036 | 0.964 | 0 | 0 | 0 | 0 | 0 | 0 | 0 | 0 |
| **BG_1_** | 0 | 0 | 0 | 0 | 0.990 | 0.010 | 0 | 0 | 0 | 0 | 0 | 0 | 0 |
| **UP** | 0 | 0 | 0 | 0 | 0 | 0.042 | 0.958 | 0 | 0 | 0 | 0 | 0 | 0 |
| **FP_p_** | 0 | 0 | 0 | 0 | 0 | 0 | 0.879 | 0.121 | 0 | 0 | 0 | 0 | 0 |
| **DN** | 0 | 0 | 0 | 0 | 0 | 0 | 0 | 0.036 | 0.001 | 0 | 0 | 0 | **0.963** |
| **BG_2_** | 0 | 0 | 0 | 0 | 0 | 0 | 0 | 0 | 0.990 | 0.010 | 0 | 0 | 0 |
| **UP** | 0 | 0 | 0 | 0 | 0 | 0 | 0 | 0 | 0 | 0.042 | 0.958 | 0 | 0 |
| **FP_s_** | 0 | 0 | 0 | 0 | 0 | 0 | 0 | 0 | 0 | 0 | 0.879 | 0.121 | 0 |
| **DN** | 0 | 0 | 0 | 0 | 0 | 0 | 0 | 0 | 0 | 0 | 0 | 0.036 | 0.964 |
| **BG_3_** | 0 | 0 | 0 | 0 | 0 | 0 | 0 | 0 | 0 | 0 | 0 | 0 | 1 |

**Table 6 HMM Emissions (standardized)**

Emissions for the all HMM’s 13 states are listed. These emissions are modeled by Gaussian distributions with the corresponding mean and standard deviation (std) shown. Note that these values are derived after standardization of raw hexamer profile coverage to the background score calculated from the training data. The BG_1_ and BG_2_ (intermediary/secondary backgrounds) were calculated (and tweaked) based on the measured BG_0_ and BG_3_ values (somewhere in between the two).

| **States** | **Arabidopsis** | | **Rice** | |
| --- | --- | --- | --- | --- |
|  | ***mean*** | ***std*** | ***mean*** | ***std*** |
| **BG_0_** | 0.370681027 | 0.145492001 | 0.530290538 | 0.231679565 |
| **UP** | -1.103074051 | 0.903519929 | -1.149144567 | 0.856341604 |
| **FP_s_** | -2.726769331 | 0.034938222 | -2.638014215 | 0.03536976 |
| **DN** | -1.359386756 | 0.969790822 | -1.290317173 | 0.832150009 |
| **BG_1_** | 0.490681027 | 0.145492001 | 0.480290538 | 0.231679565 |
| **UP** | -1.103074051 | 0.903519929 | -1.149144567 | 0.856341604 |
| **FP_p_** | -2.726769331 | 0.034938222 | -2.638014215 | 0.03536976 |
| **DN** | -1.359386756 | 0.969790822 | -1.290317173 | 0.832150009 |
| **BG_2_** | 0.490681027 | 0.145492001 | 0.480290538 | 0.231679565 |
| **UP** | -1.103074051 | 0.903519929 | -1.149144567 | 0.856341604 |
| **FP_s_** | -2.726769331 | 0.034938222 | -2.638014215 | 0.03536976 |
| **DN** | -1.359386756 | 0.969790822 | -1.290317173 | 0.832150009 |
| **BG_3_** | 0.629707395 | 0.228739766 | 0.476770207 | 0.27267674 |

**Table 7 Motifs from enriched hexamers**

Motifs generated after clustering the IR and IE enriched hexamers exhibiting a footprint across in leaf samples in both species. Motif logos were generated using the weblogo tool. In the table, these motifs are grouped based on the type of event (IR and IE) they are enriched in and part of the event from which their respective hexamers were found (5’ exon, intron, and 3’ exon).

| **Event type** | **Part of event** | **Consensus sequence** | **Motif logo** |
| --- | --- | --- | --- |
| IR | 5’ Exon | CGCCG | 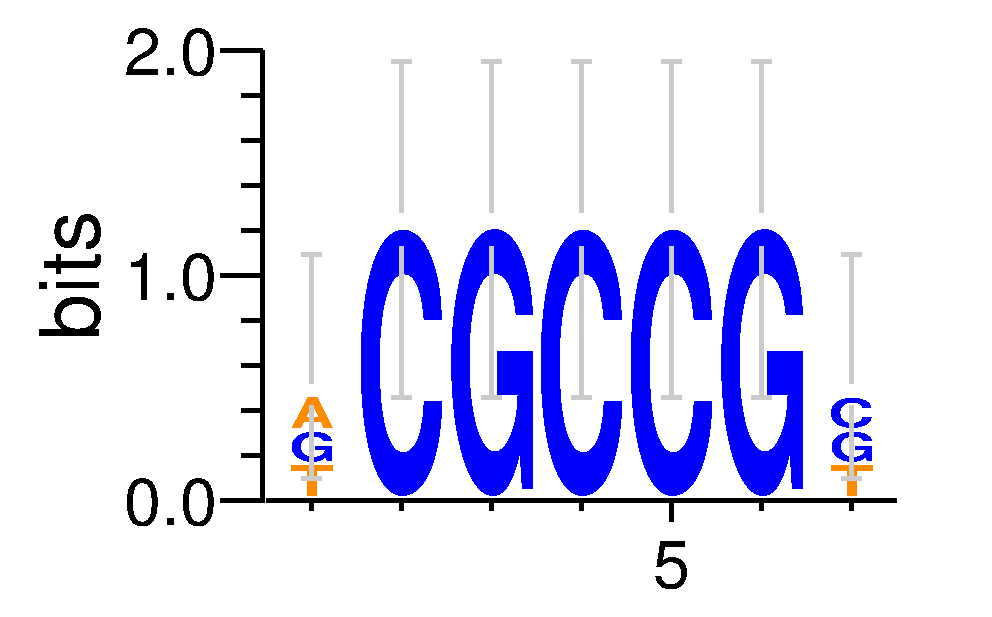 |
|  |  | (G/C)(G/C)GCGG | 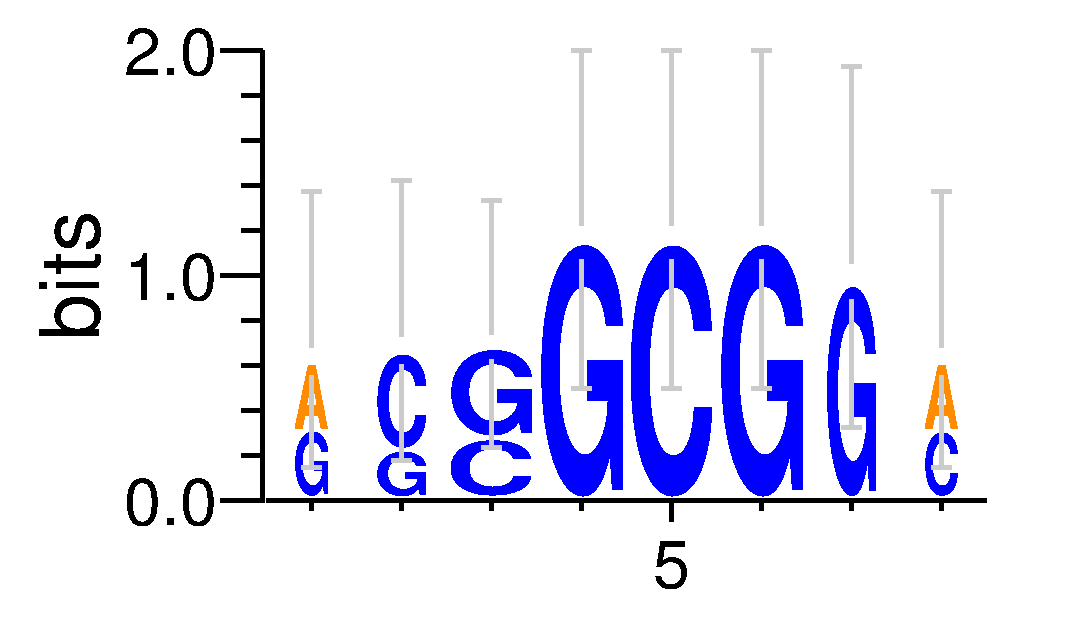 |
|  |  | (A/G)T(C/T)(G/T)(C/G)A | 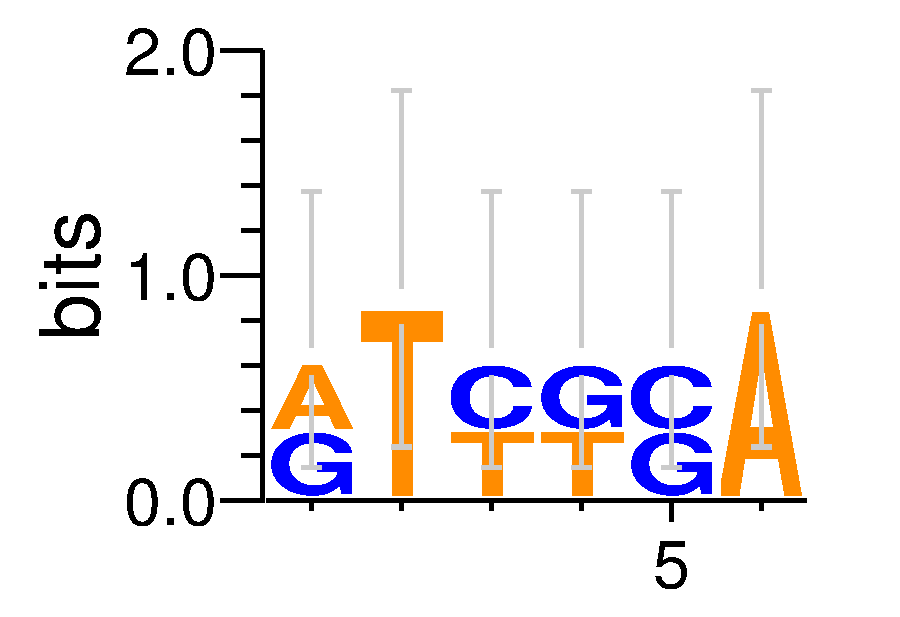 |
|  | 3’ Exon | AAAA | 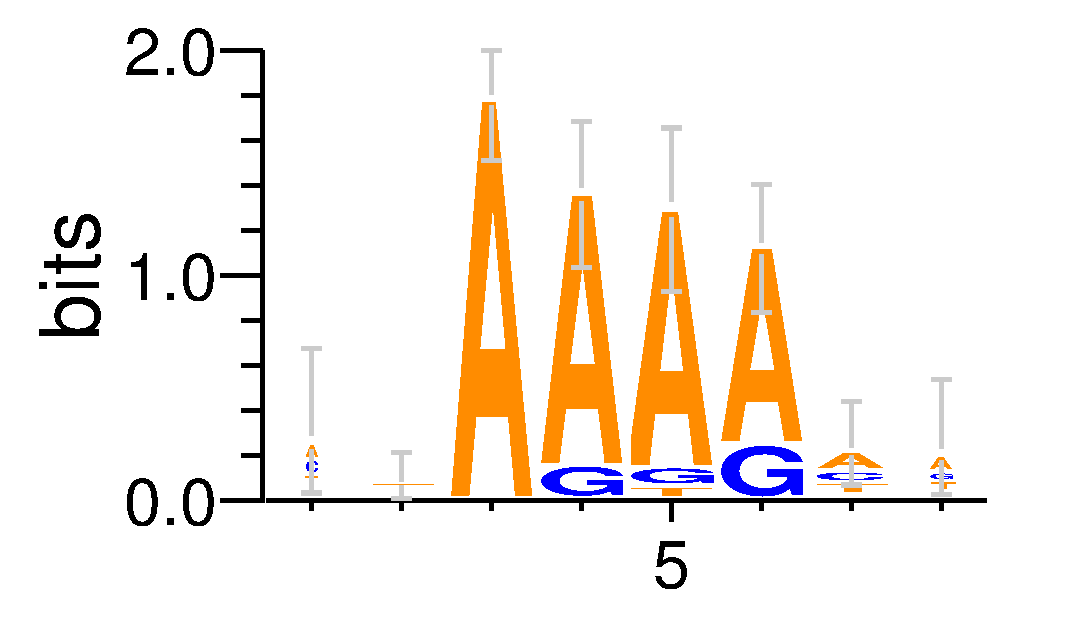 |
|  |  | AAATT | 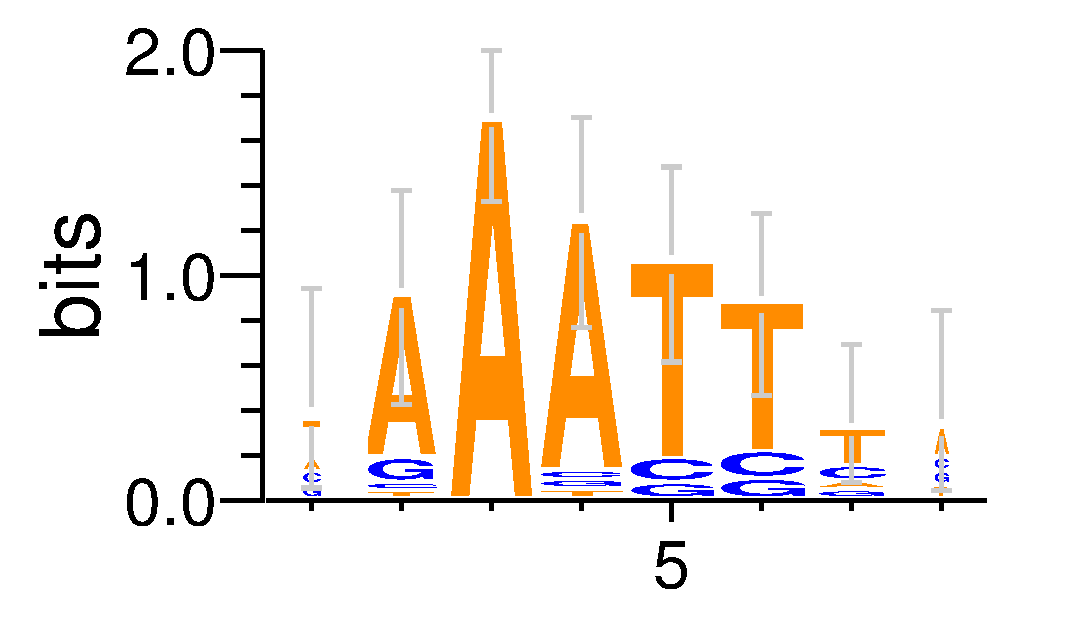 |
|  |  | CCGACG | 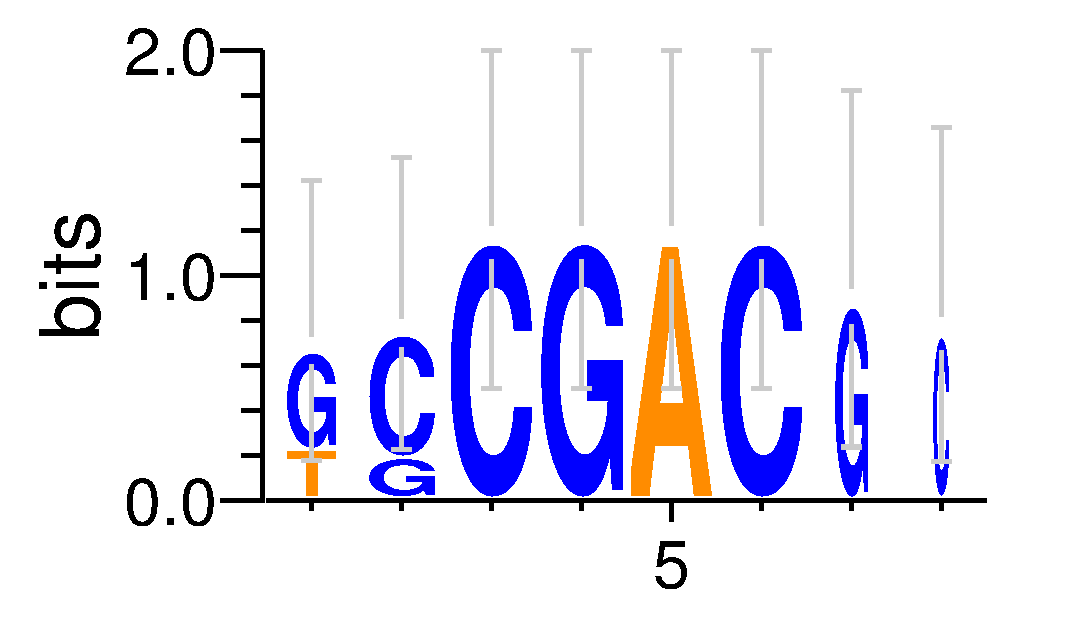 |
|  |  | CGCxCG | 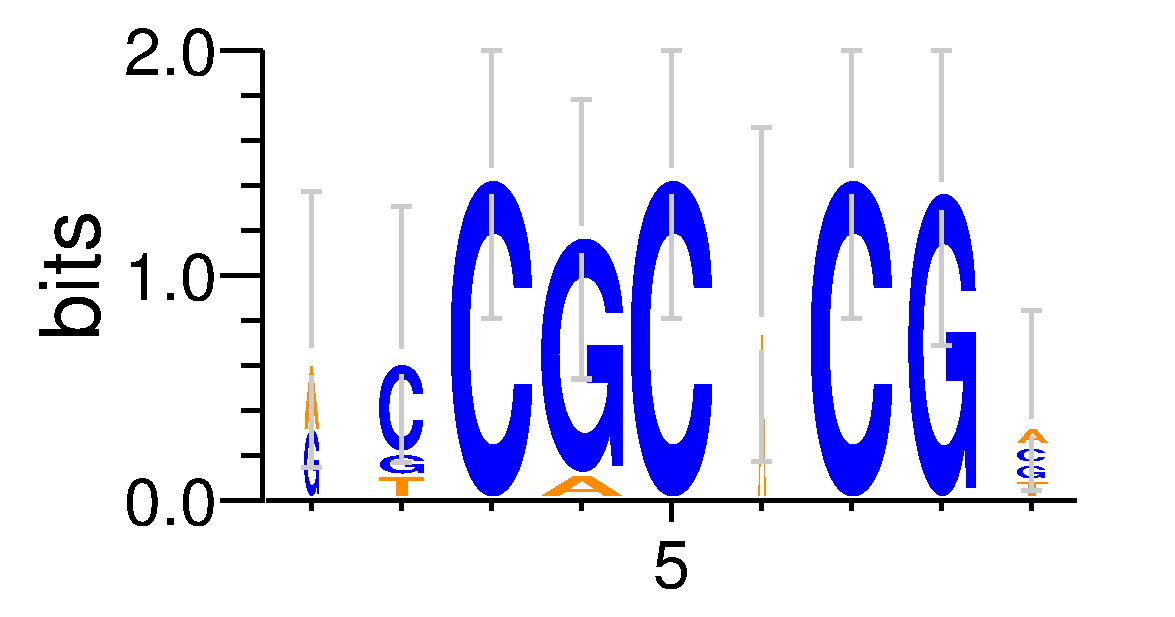 |
|  |  | (C/A)TTT | 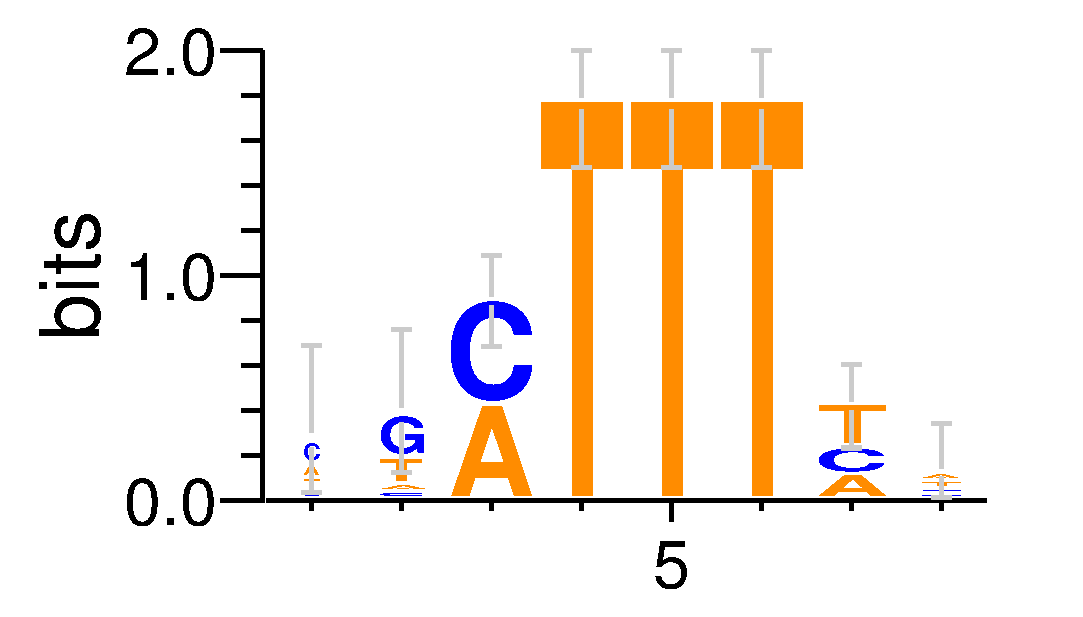 |
|  |  | CGGCG | 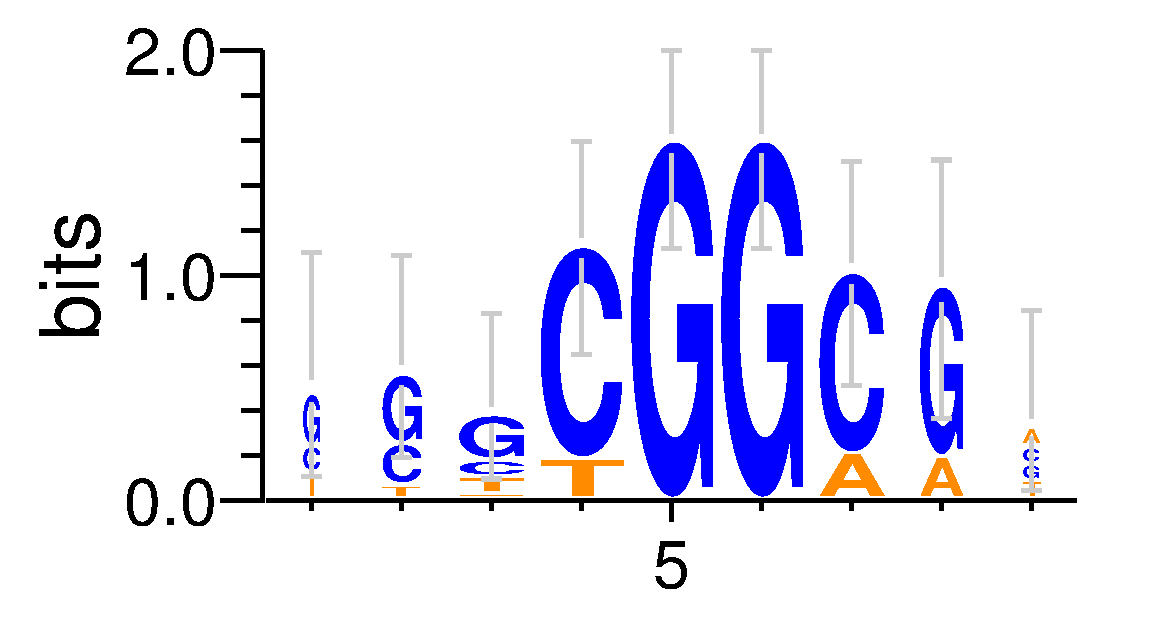 |
|  |  | GxTTT | 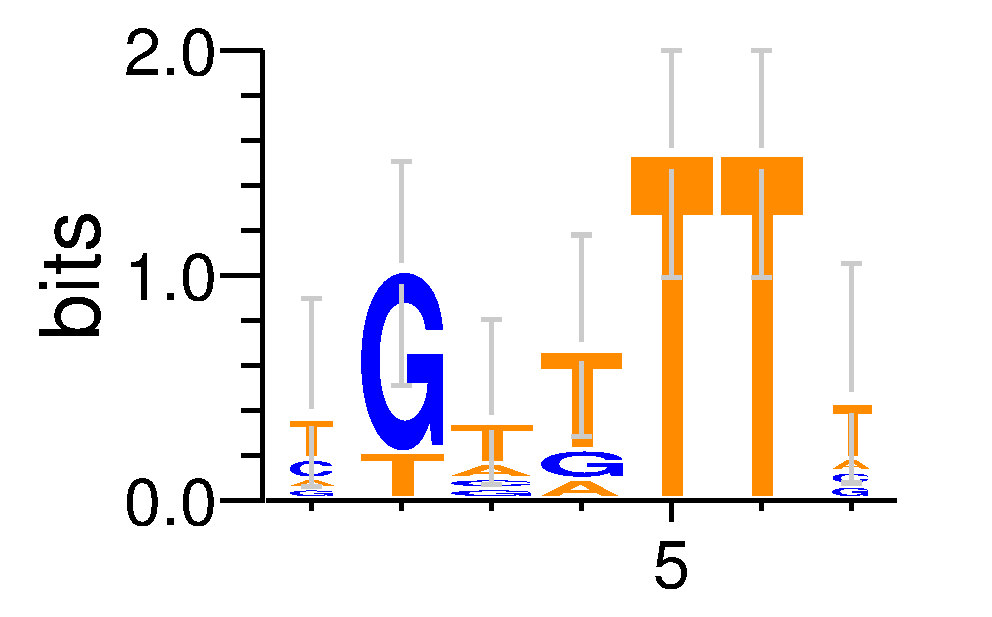 |
|  |  | (T/G)AAA | 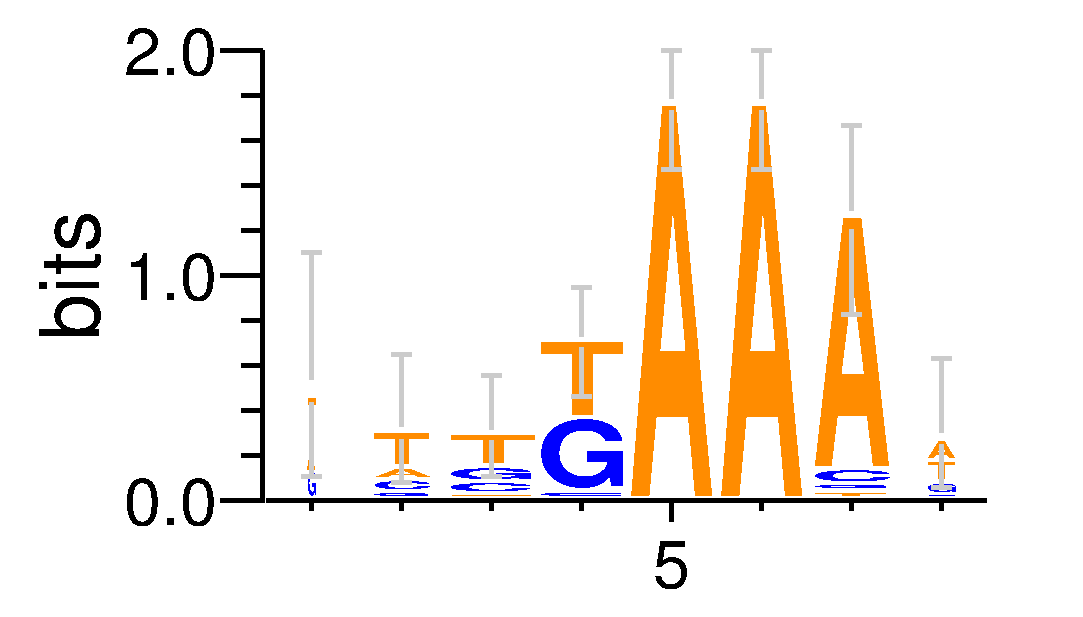 |
|  |  | (G/T)T(C/T)(C/G)(G/A) | 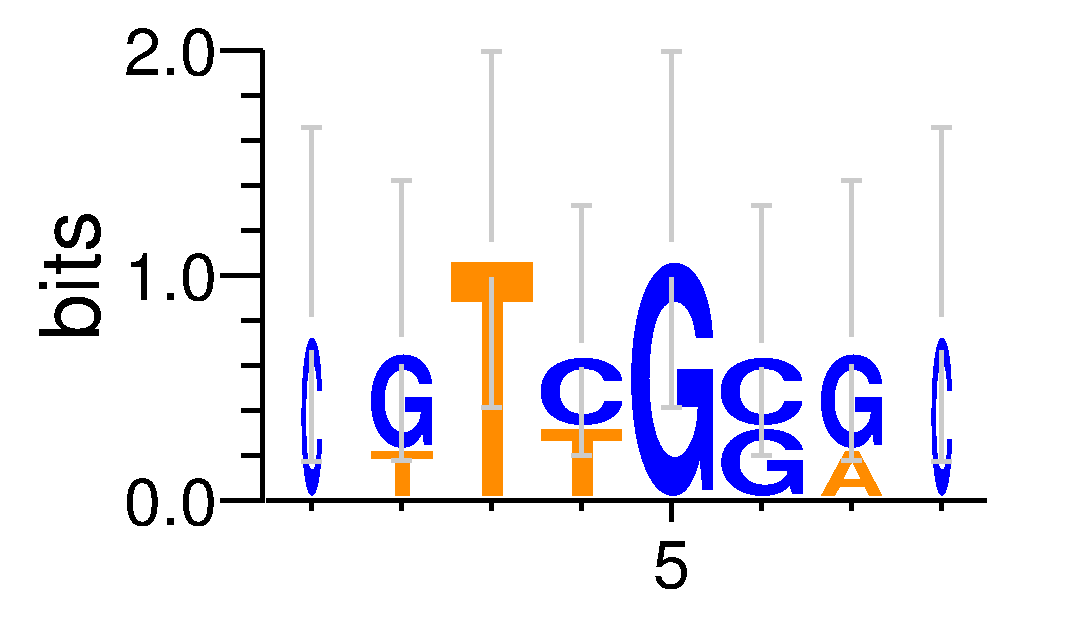 |
|  |  | TTT(C/T) | 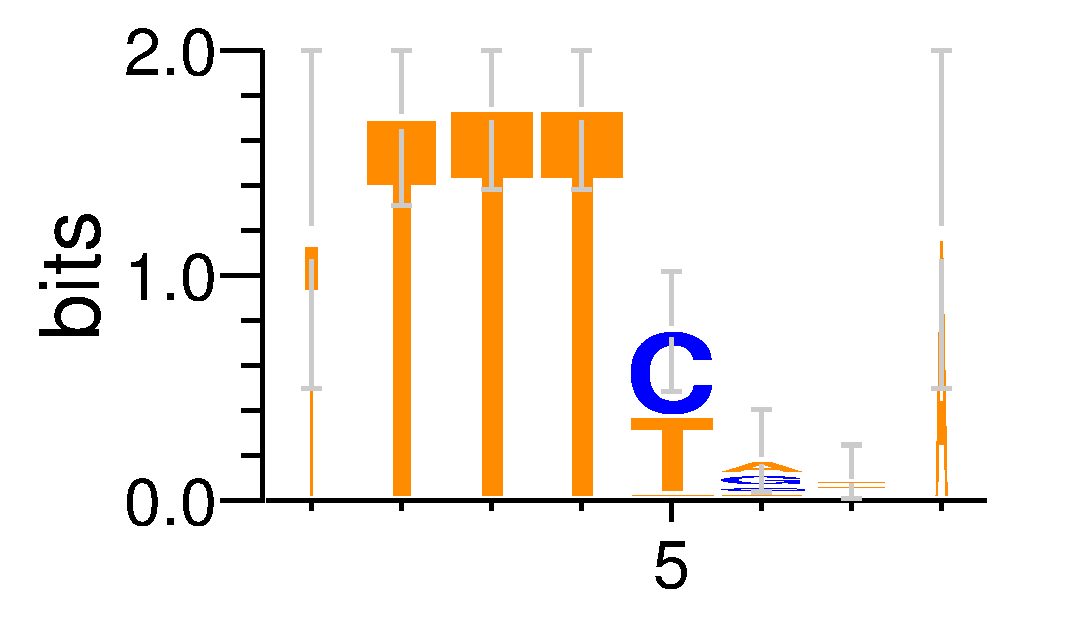 |
| IE | Intron | TTAA(T/A)(T/A) | 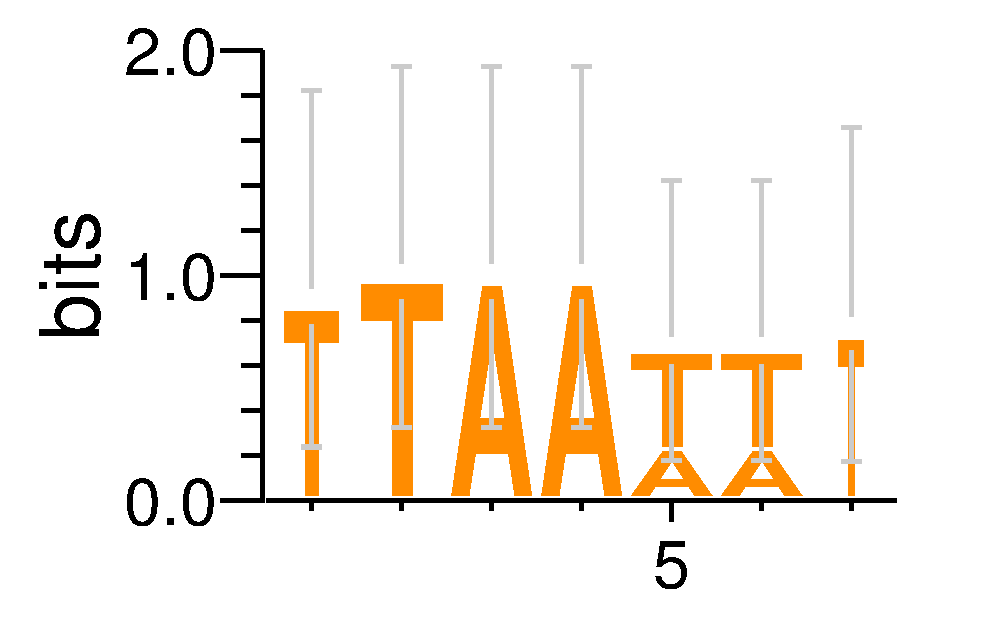 |
|  |  | T(T/A)TTT(A/T) | 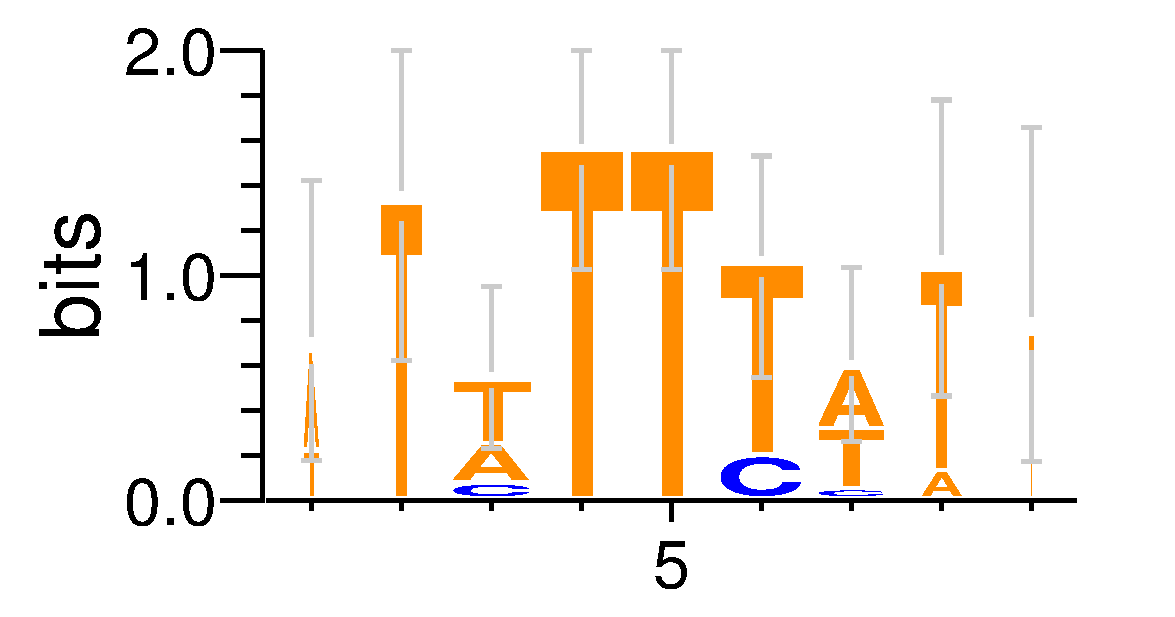 |

**Table 8 Hexamers and Cistrome overlap summary**

The overlap stats between all significantly enriched arabidopsis IR/IE hexamers and transcription factor motifs from Plant Cistrome Database are summarized below. The actual overlaps are provided in the Additional file 3.

| **AS Type** | **Part** | **Total Motifs** | **Total Hexamers** | **Overlapping Hexamers** |
| --- | --- | --- | --- | --- |
| **IR** | 5' Exon | 410 | 13 | 6 |
|  | Intron |  | 2 | 1 |
|  | 3' Exon |  | 246 | 80 |
| **IE** | Intron |  | 19 | 9 |

**References**

1. Zhang, W., Zhang, T., Wu, Y., Jiang, J.: Genome-wide identification of regulatory DNA elements and protein-binding footprints using signatures of open chromatin in arabidopsis. Plant Cell24, 2719–31(2012)
2. Zhang, W., Wu, Y., Schnable, J.C., Zeng, Z., Freeling, M., Crawford, G.E., Jiang, J.: High-resolution mapping of open chromatin in the rice genome. Genome Research22, 151–62 (2012)
3. Zemach, A., Kim, M.Y. et al.: The Arabidopsis Nucleosome Remodeler DDM1 Allows DNA Methyltransferase to Access H1-Containing Heterochromatin. Cell153, 193–205 (2013)
4. Chodavarapu, R.K., Feng, S. et al.: Transciptome and methylome interactions in rice hybrids. PNAS109, 12040–45 (2012)
